# Use of Hemoadsorption and Continuous Venovenous Hemodialysis With Enhanced Middle Molecule Clearance in Drug-Induced Rhabdomyolysis

**DOI:** 10.1155/crcc/3968057

**Published:** 2025-01-20

**Authors:** Sebastian Hafner, Johannes Reins, Christoph Baader, Florian Balling, Sebastian Eff

**Affiliations:** ^1^Department of Anesthesiology and Intensive Care Medicine, Sana Klinikum Landkreis Biberach, Biberach, Germany; ^2^Department of Anesthesiology and Intensive Care Medicine, Kreiskliniken Günzburg-Krumbach, Krumbach, Germany

**Keywords:** antidepressants, antipsychotics, CVVHD, CytoSorb, drug-induced rhabdomyolysis, hemoadsorption, metabolic acidosis, myoglobin, statins

## Abstract

Drug-induced rhabdomyolysis has become increasingly prevalent due to the rising use of medications such as statins, antidepressants, and antipsychotics. These can lead to muscle cell destruction and the release of myoglobin, potentially causing kidney damage. Recent advancements include the use of CytoSorb hemoadsorption as a promising therapy to remove myoglobin and other potentially toxic substances from the bloodstream. A 47-year-old male with a complex medical history presented with weakness, pain, and dizziness. Lab results indicated severe rhabdomyolysis, most likely of medication-induced etiology. He developed acute kidney injury (AKI) and underwent continuous venovenous hemodialysis (CVVHD) combined with CytoSorb hemoadsorption. Despite initial stabilization, rhabdomyolysis parameters surged, necessitating the use of an additional high-flux filter with enhanced middle molecule clearance. CytoSorb therapy was administered for nine consecutive sessions, resulting in decreased creatine kinase (CK) and myoglobin levels. Due to persistent kidney injury, the patient required permanent dialysis and was transferred to a kidney disease center. This case highlights the complexity and severity of drug-induced rhabdomyolysis with hemoadsorption playing a pivotal role in reducing myoglobin levels and improving the patient's condition. Combining hemoadsorption and filters with enhanced middle molecule clearance holds even more promise for improved myoglobin removal.

## 1. Background

Rhabdomyolysis, characterized by the rapid breakdown of skeletal muscle fibers and release of intracellular contents into the bloodstream, is a significant medical emergency [1]. Drug-induced toxicity, an increasingly recognized cause, has risen with the use of medications like statins, antidepressants, and antipsychotics [2, 3]. This condition typically results from direct muscle toxicity, impaired cellular energy production, or immune-mediated responses, leading to myoglobin release, which can damage the kidneys and cause myoglobinuric acute kidney injury (AKI) [2, 4]. Clinically, it presents with muscle pain, weakness, dark urine, and elevated serum creatine kinase (CK) and myoglobin levels. Prompt diagnosis and intervention are crucial to prevent severe renal dysfunction and multiorgan failure [5]. The therapeutic approach to drug-induced rhabdomyolysis primarily involves discontinuing the triggering medication, fluid resuscitation to maintain renal perfusion, alkalinization of urine, and addressing any electrolyte disturbances. In severe cases, renal replacement therapy may be required [5].

Recent advancements in the management of rhabdomyolysis have introduced CytoSorb hemoadsorption as a promising adjunctive therapeutic option [6]. CytoSorb, a novel extracorporeal hemoadsorption device, is indicated for the removal of elevated levels of circulating cytokines, myoglobin, and other (toxic) substances. This innovative approach holds the potential to reduce the severity of rhabdomyolysis and its associated complications by effectively clearing toxic substances from the bloodstream.

In this report, we present the case of drug-induced rhabdomyolysis with a complex medical history, emphasizing the clinical challenges posed by this condition and the utilization of hemoadsorption as a pivotal component of the therapeutic strategy.

## 2. Case Presentation

**Figure 1:**
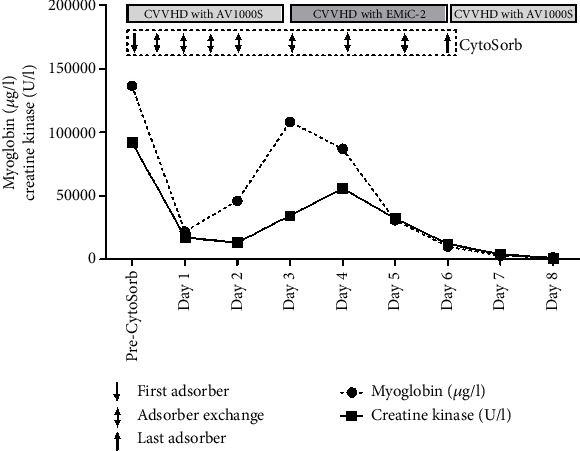
Course of myoglobin and creatine kinase as well as treatment modalities during the critical phase of a patient with drug-induced rhabdomyolysis and severe metabolic acidosis.

A 47-year-old male patient with a complex medical history was admitted to our hospital via the emergency department. His known medical conditions included arterial hypertension, nicotine abuse, hyperlipoproteinemia, non-insulin–dependent diabetes mellitus, peripheral vascular disease, a history of myocardial infarction with subsequent stenting, and a past diagnosis of depressive episodes managed with antidepressive drugs. He also had a history of excessive chronic alcohol consumption. Upon arrival at the emergency room, the patient complained of a history of increasing weakness, extremity pain, and dizziness for several days before admission, which culminated in a fall, resulting in a left parietal head laceration. However, he exhibited stable hemodynamic conditions. Venous blood gas analysis revealed metabolic acidosis with a base excess of −13 mmol/L and hyperkalemia (7.5 mmol/L). A thorough assessment by the surgical team determined that treatment for the head laceration was not immediately required. Cranial CT examination ruled out acute hemorrhage, ischemia, or space-occupying lesions. Subsequently, the patient was admitted to the intensive care unit (ICU) for further evaluation and management. Laboratory tests indicated significantly elevated retention parameters, along with excessively increased CK (92,047 *μ*g/L) and myoglobin levels (136,554 *μ*g/L) (Table 1 and Figure 1). Suspecting rhabdomyolysis, most likely medication induced, the administration of sertraline, ezetimibe, and rosuvastatin was promptly discontinued. Conservative treatment options such as intravenous fluids and urine alkalinization were not feasible due to the patient's severe AKI with oligo-anuria, hyperkalemia, hydropic decompensation, and metabolic acidosis. These conditions necessitated immediate dialysis, as forced diuresis or urine alkalinization was no longer possible. Therefore, continuous venovenous hemodialysis (CVVHD) with regional citrate anticoagulation (multiFiltratePRO, Fresenius Medical Care, Bad Homburg, Germany) was initiated in combination with CytoSorb hemoadsorption (CytoSorbents Europe GmbH, Berlin, Germany) applied in prehemofilter position for 7 consecutive days (total of nine adsorbers used). To avoid saturation of the adsorber in the early treatment phase, the adsorber was changed every 12 h on Days 1 and 2 and every 24 h from Day 3 onwards. After rapid initial stabilization under this combination, a renewed surge in rhabdomyolysis parameters occurred, which led to the replacement of the AV1000S Ultraflux dialysis filter with an Ultraflux EMiC2 filter with enhanced middle molecule clearance (Fresenius Medical Care, Bad Homburg, Germany) from Day 3 to Day 6 (Figure 1). Following consistent decreases in CK and myoglobin values, CytoSorb therapy could be discontinued after a total of nine consecutive sessions (Figure 1). Nevertheless, the patient remained anuric, necessitating ongoing dialysis throughout his ICU stay. Due to the onset of hydropic decompensation at the time of admission to the ICU, intermittent noninvasive ventilation (NIV) and high-flow nasal cannula (HFNC) therapy were employed. Fortunately, stable respiratory parameters were achieved following ongoing negative fluid balance adjustments.

After 13 days of intensive care therapy, the patient was transferred to a kidney disease center for further therapy and intermittent dialysis. The patient's cardiocirculatory status remained stable; in addition, the patient required 3 L of oxygen via nasal cannula.

## 3. Discussion

The case of drug-induced rhabdomyolysis in this 47-year-old male patient illustrates the complexity and severity of this condition, which can be triggered by various medications and necessitates comprehensive management.

Drug-induced rhabdomyolysis can result from the toxic effects of certain medications on skeletal muscle cells, involving mechanisms such as direct muscle toxicity, mitochondrial dysfunction, or immune-mediated responses [7]. In this case, the patient's medication history included sertraline, ezetimibe, and rosuvastatin, all linked to rhabdomyolysis in literature reports, particularly statins like rosuvastatin, which can impair mitochondrial function and lead to muscle injury [8]. Moreover, both the patient's history of excessive chronic alcohol consumption and the fall could have also contributed to the development of rhabdomyolysis. Chronic alcohol use is a known risk factor for rhabdomyolysis due to its toxic effects on muscle cells, potentially leading to muscle breakdown. Additionally, trauma or prolonged immobilization following a fall may exacerbate muscle injury and increase the risk of rhabdomyolysis. These factors, alongside the drug-induced component, likely played a role in the pathogenesis of this patient's condition and should be considered as potential contributing mechanisms.

Clinical presentation typically involves muscle pain, weakness, dark urine, and elevated serum CK and myoglobin levels. The patient exhibited typical symptoms and exceptionally high CK and myoglobin levels, indicating extensive muscle damage. Rhabdomyolysis can lead to renal complications, necessitating close monitoring of the renal function. Elevated myoglobin levels can precipitate in renal tubules, potentially causing myoglobinuric AKI, known as “crush kidney.” Therefore, in rhabdomyolysis, rapid myoglobin clearance reduces the risk of myoglobin-induced renal injury and other downstream effects.

From our point of view, CytoSorb hemoadsorption played a notable role in this patient's treatment. CytoSorb is an extracorporeal hemoadsorption device with a substantial surface area for adsorption and removal of circulating toxins, including myoglobin and inflammatory mediators. Blood passes through the adsorber, allowing toxins to bind and be removed from the circulation, offering a dynamic and adaptable means of addressing the diverse toxicities associated with rhabdomyolysis.

Initiating CytoSorb therapy led to a significant reduction in myoglobin levels, likely contributing to the patient's overall improvement. Of note, the combination with the EMiC-2 filter from Day 3 onwards might be an interesting way to increase the removal capacity for myoglobin even further.

High cut-off (HCO) dialysis filters have shown potential in removing larger molecules such as myoglobin, which cannot be sufficiently cleared by standard dialysis filters. HCO filters allow for the clearance of middle molecules, including myoglobin (17 kDa), owing to their larger pore size. In our case, we utilized the Ultraflux EMiC2 filter, which has enhanced middle molecule clearance capabilities. While CytoSorb hemoadsorption is recognized for its ability to adsorb myoglobin, combining it with a filter specifically designed for middle molecule removal may provide a synergistic effect, enhancing the overall clearance.

Several studies suggest that HCO dialysis is effective in reducing myoglobin levels in patients with rhabdomyolysis [9], particularly in cases of AKI. HCO filters facilitate greater permeability for myoglobin, leading to more efficient removal from the bloodstream, which may help prevent myoglobin-induced renal injury. This method of enhanced dialysis may thus provide an effective adjunct to hemoadsorption.

In our case, the use of the EMiC2 filter in combination with CytoSorb hemoadsorption could have increased the elimination capacity for myoglobin, leading to a faster decline in myoglobin levels. This combination of modalities targets both adsorption and diffusion, providing a broader spectrum of removal of toxic middle molecules.

It is important to consider that the patient's rhabdomyolysis could have improved spontaneously, which may have contributed to the observed reduction in myoglobin levels. Rhabdomyolysis is often self-limiting once the causative agent (e.g., medication) is discontinued, and muscle breakdown slows down as the condition resolves. Hence, while we attribute much of the myoglobin clearance to the combination of CytoSorb hemoadsorption and HCO dialysis, it is possible that the natural course of recovery from rhabdomyolysis also played a role in the observed decline in myoglobin levels. We acknowledge this possibility and emphasize that further studies are required, which include the analysis of myoglobin concentration pre- and postfilter as well as pre- and postadsorber, to clearly delineate the contribution of each modality to the observed improvements in such cases.

We initially considered using continuous venovenous hemofiltration (CVVH), as it generally removes myoglobin more effectively due to its convective clearance mechanism, potentially offering better protection against myoglobin-induced AKI [10]. However, at the time of treatment, only continuous venovenous hemodialysis with an HCO filter (CVVHD-HCO) was available at our hospital. Given these limitations, we opted for CVVHD-HCO combined with CytoSorb hemoadsorption. This approach allowed for both middle molecule clearance through the HCO filter and the additional toxin removal offered by CytoSorb, providing an effective alternative for myoglobin removal in this case.

An additional benefit of CytoSorb in removing drugs like sertraline, ezetimibe, and rosuvastatin is conceivable in principle. However, no specific data exists to confirm its effectiveness in eliminating these particular drugs. While CytoSorb is effective in adsorbing a range of middle molecules, its role in removing these medications remains uncertain.

Unfortunately, despite the therapeutic interventions, the patient's kidney function did not recover, and he remained dependent on dialysis. However, the intervention led to a rapid and significant reduction of the extremely high myoglobin concentration, which is critical in cases of rhabdomyolysis. In other patients, timely reduction of myoglobin levels could potentially help preserve renal function and prevent progression to end-stage renal disease, highlighting the importance of early and aggressive myoglobin removal in similar clinical scenarios.

These results are in line with other published data. One of the largest retrospective observational case series to date investigated 43 patients with anuric AKI with the need for renal replacement therapy, who additionally received CytoSorb therapy because of rhabdomyolysis resulting from septic shock, trauma, and hypovolemic or cardiogenic shock [11]. Patients were divided into those with persistent rhabdomyolysis (ongoing CK and myoglobin production from ongoing muscle injury) and those in which rhabdomyolysis was resolving. Overall, myoglobin levels decreased by 29% with a higher reduction of 38% seen in patients without ongoing rhabdomyolysis. Since all patients had anuric AKI, renal elimination of myoglobin cannot be assumed and, in the authors' view, the reduction of myoglobin was largely due to the use of CytoSorb and not due to the high-flux dialyzer [11]. The authors conclude that myoglobin removal with the CytoSorb integrated into a dialysis circuit can be recommended for clinical routine due to its existing clinical approval, ease of use, and absence of side effects [11].

The study by Albrecht et al. highlights the effectiveness of CytoSorb hemoadsorption in severe rhabdomyolysis [6]. Their findings showed that CytoSorb reduced myoglobin levels significantly faster than standard treatment. Patients in the CytoSorb group had a myoglobin reduction of 76% within 5 min, which dropped to 10% at 8 h, compared to minimal reductions in the control group.

Our case supports these findings as we observed a significant decrease in myoglobin levels. In addition, due to saturation, it was necessary to replace the CytoSorb adsorber every 12 hours on Days 1 and 2 (from Day 3 onwards every 24 h) to maintain effective myoglobin elimination, consistent with results from Albrecht et al. [6], ensuring sustained myoglobin reduction, which highlights the importance of regular adsorber exchange for optimal treatment outcomes.

The use of CytoSorb hemoadsorption in severe rhabdomyolysis has shown significant efficacy in various studies. Hui et al. successfully treated pediatric rhabdomyolysis–associated AKI with CytoSorb and continuous renal replacement therapy (CRRT) [12], and Zitoune et al. reported positive outcomes in acute tricyclic antidepressant poisoning with severe rhabdomyolysis using ECMO and CytoSorb [13].

Dilken et al. and Moresco et al. both documented rapid reductions in CK and myoglobin levels using CytoSorb [14, 15]. A retrospective analysis by Rauch et al. highlighted its role in kidney replacement therapy and prevention of rhabdomyolysis-associated AKI [16]. Suefke et al. supported CytoSorb's utility in infection-associated rhabdomyolysis [17]. Gräfe et al. even demonstrated in an observational study that the use of CytoSorb might positively affect renal recovery in patients with severe rhabdomyolysis [18].

Further, Mikacic et al. and Immohr et al. showed CytoSorb's effectiveness in treating rhabdomyolysis due to exertional heat stroke and postheart transplantation, respectively [19, 20]. Padiyar et al. and Wiegele and Krenn documented successful outcomes in pediatric cases and infectious disease–associated rhabdomyolysis [21, 22]. All these studies and case reports affirm CytoSorb's versatility and efficacy in rapidly managing severe rhabdomyolysis and preventing renal damage.

In conclusion, drug-induced rhabdomyolysis poses a multifaceted clinical challenge, especially when extensive muscle damage, renal impairment, and other complications occur. This case emphasizes early diagnosis, immediate cessation of possibly triggering medication, and aggressive supportive care. Integrating CytoSorb hemoadsorption into the therapeutic approach holds promise for improving outcomes by efficiently removing elevated levels of myoglobin and cytokines from the circulation. Further research and clinical experience are needed to evaluate CytoSorb's role in managing rhabdomyolysis and other critical conditions.

## Figures and Tables

**Table 1:** Relevant laboratory and clinical parameters during the clinical course.

**Parameter**	**Unit**	**Reference**	**Pre-CytoSorb** ^**a**^	**Day 1**	**Day 2**	**Day 3**	**Day 4**	**Day 5**	**Day 6**	**Day 7**	**Day 8**
Sodium	mmol/L	135–145	121	131	139	137	135	138	138	137	143
Potassium	mmol/L	3.50–5.10	7.51	4.27	4.40	5.12	4.64	4.24	4.24	4.14	5.01
Phosphate	mmol/L	0.87–1.45		1.98		1.36	1.52	1.27	1.07	1.07	1.03
Lactate dehydrogenase	U/L	< 250	3248	1350	1082	1222	1740	1050	688	560	486
Urea	mg/dL	< 50	183	83	45	35	35	38	35	36	38
pH		7.37–7.45	7.29	7.42	7.47	7.47	7.45	7.47	7.43	7.45	7.51
Base excess	mmol/L	−2.0 to 3.0	−14.1	−5.7	1.2	1.4	0	1.7	0.4	4.6	10.9
Lactate	mmol/L	< 1.80	1.7	1.5	1.3	1.0	1.0	1.1	1.2	2.4	1.8
pCO_2_	mmHg	35–46	21.7	26.6	34.1	34.4	34.5	34.5	37.4	41.8	43.9
HCO_3_^−^	mmol/L	21–26	13.6	19.7	25.4	25.6	24.5	25.9	24.8	28.5	34.6
Fluid balance	mL	n.a.	n.a.	1550	2220	2500	1335	835	645	−100	−1000
Diuresis	mL/day	n.a.	n.a.	60	20	200	45	55	20	0	0

*Note:* The laboratory values for Days 1–8 were collected each morning at 5: 00 a.m. during the course of treatment.

^a^Pre-CytoSorb values were collected upon the patient's admission to the intensive care unit (ICU), just before starting the treatment with CytoSorb.

## Data Availability

Data sharing is not applicable to this article as no additional data were created or analyzed.
